# Supplementary figures and images for: Ex vivo model of herpes simplex virus type I dendritic and geographic keratitis using a corneal active storage machine
Source: PLoS One. 2020 Jul 22;15(7):e0236183. doi: 10.1371/journal.pone.0236183 (PMC7375596; doi:10.1371/journal.pone.0236183)

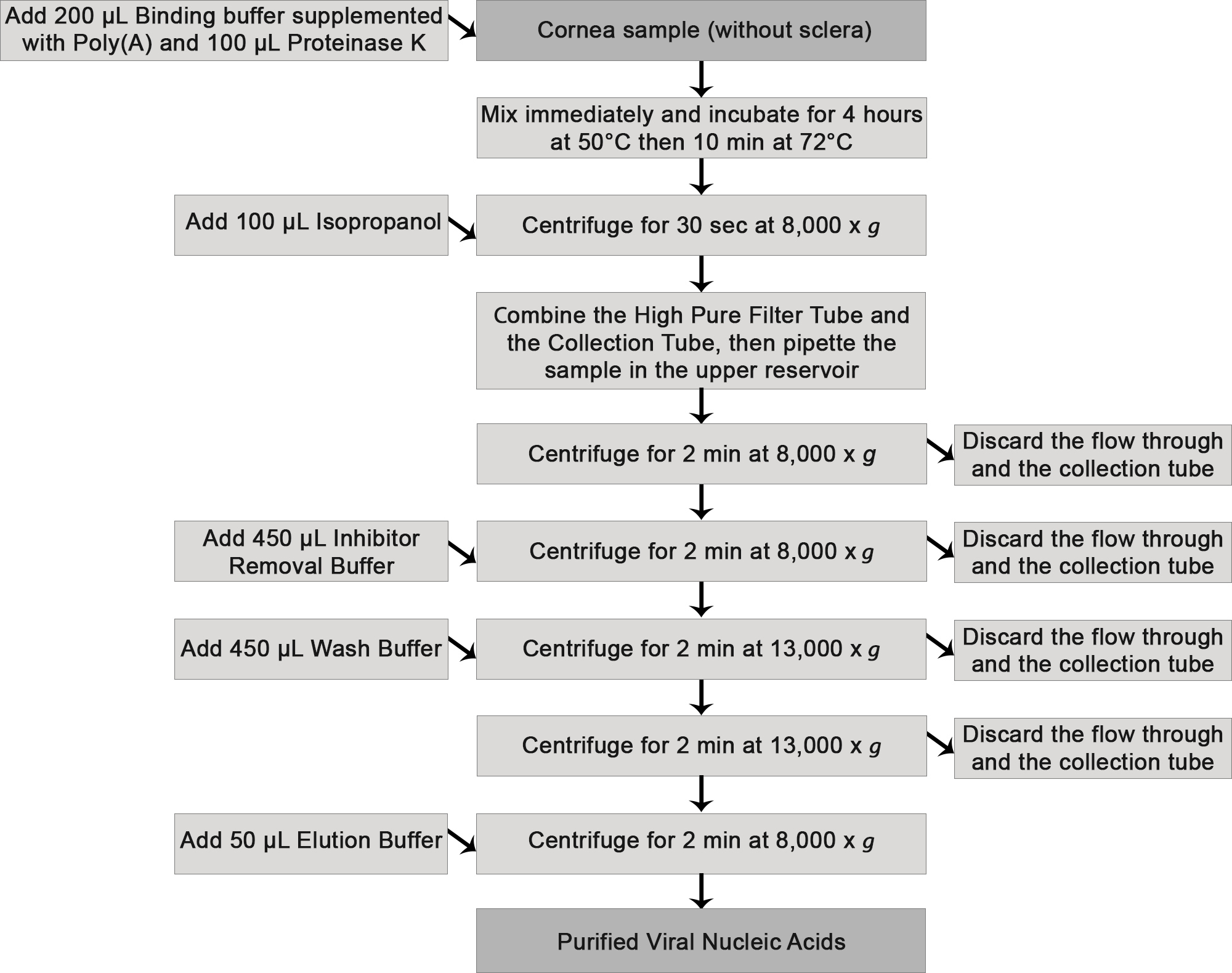

Supplement: S1 Fig — (TIF) [file pone.0236183.s001.tif]

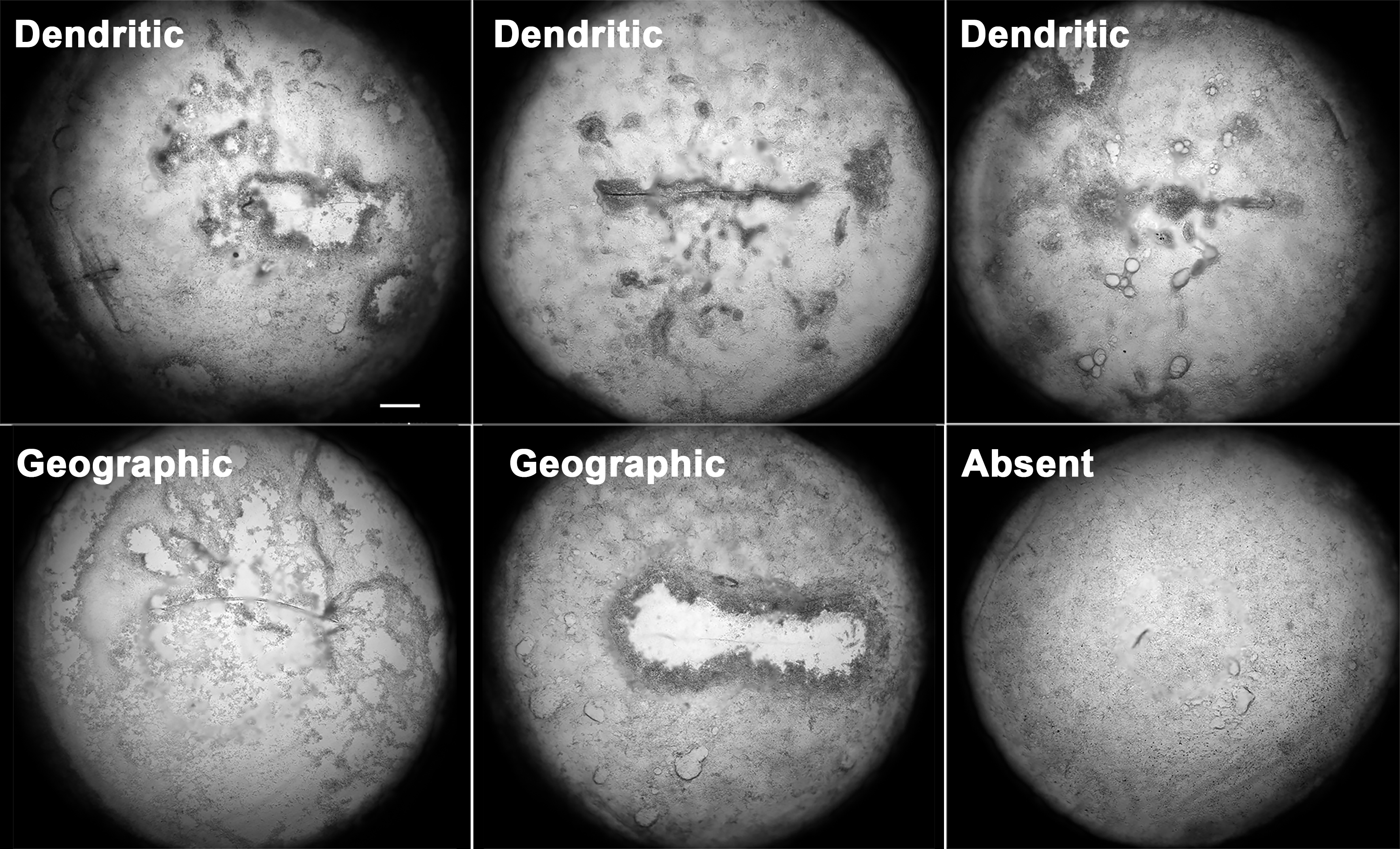

Supplement: S2 Fig — The infection pattern is shown at the top of each picture. Scale bar: 1000μm. (TIF) [file pone.0236183.s002.tif]
