# Supplementary material for: Ex vivo model of herpes simplex virus type I dendritic and geographic keratitis using a corneal active storage machine
Source: PLoS One. 2020 Jul 22;15(7):e0236183. doi: 10.1371/journal.pone.0236183 (PMC7375596; doi:10.1371/journal.pone.0236183)
Supplement: S1 Table — (DOCX) [file pone.0236183.s003.docx]

**Table S1.** Primary antibodies used for immunostaining on flat-mounted corneas and on cross-sections.

| Target Protein | Role (Expected Cell Compartment) | Source | Isotype | Reference and Manufacturer | Application, fixative (dilution) |
| --- | --- | --- | --- | --- | --- |
| Glycoprotein B | Infected cells (plasmic membranes) | mouse | IgG2B | SAB4700766 (Sigma) | IF-FM/0.5% PFA or methanol (1/300) and IF-CS (1/500) |
| e-Cadherin | Epithelial cells (all plasmic membranes) | mouse | IgG1 | 33400 (Invitrogen) | IF-FM/methanol (1/300), IF-CS (1/500) |
| Cytokeratin 3 (K3) | Differentiated superficial corneal epithelial cells (cytoplasmic) | mouse | IgG1 | sc-80000 (Santa Cruz) | IF-FM/0.5% PFA (1/300), IF-CS (1/500) |
| DSC2/3 | Intermediate and superficial epithelial cells (plasmic membranes) | mouse | IgG1 | sc-53485 (Santa Cruz) | IF-FM/0.5% PFA (1/300), IF-CS (1/500) |
| Laminin-5 | Epithelial basement membrane | rabbit | IgG | ab14509 (Abcam) | IF-CS (1/1000) |
| Neurofilament-L (NF-L) | Neurofilament light polypeptide | rabbit | IgG | 2837 (Cell Signaling Technology) | IF-FM/0.5% PFA (1/300) |

IF-FM: Immunofluorescence-immunostaining on flat-mounted cornea; IF-CS: Immunofluorescence- immunostaining on cross section. PFA: paraformaldehyde
