# Supplementary material for: Ex vivo model of herpes simplex virus type I dendritic and geographic keratitis using a corneal active storage machine
Source: PLoS One. 2020 Jul 22;15(7):e0236183. doi: 10.1371/journal.pone.0236183 (PMC7375596; doi:10.1371/journal.pone.0236183)
Supplement: S2 Table — (DOCX) [file pone.0236183.s004.docx]

**Table S2.** Characteristics of the pairs of corneas, and main results obtained.

| Sex/  Donor age (years) | Group | Storage duration before rehabilitation in the ASM (days) | Time of epithelial rehabilitation step in ASM (days) | HSV-1 | Duration of infection (days) | Infection pattern |
| --- | --- | --- | --- | --- | --- | --- |
| M/93 | ASM | 8 | 16 | + | 6 | Dendritic |
|  | OC |  |  | + | 6 | Buds and ulcer |
| F/83 | ASM | 9 | 16 | + | 3 | Geographic |
|  | OC |  |  | + | 3 | Buds and ulcer |
| F/77 | ASM | 21 | 14 | + | 5 | Dendritic |
|  | OC |  |  | + | 5 | Buds and ulcer |
| M/76 | ASM | 21 | 12 | + | 5 | Dendritic |
|  | OC |  |  | + | 5 | No epithelium |
| M/81 | ASM | 29 | 17 | + | 5 | Geographic |
|  | OC |  |  | + | 5 | No epithelium |
| M/83 | ASM | 51 | 16 | + | 7 | Absent |
|  | OC |  |  | + | 7 | Buds and geographic ulcer |
| M/89* | ASM | 35 | 18 | + | 4 | Dendritic |
| M/48 | ASM | 61 | 19 | - |  | Absent |
|  | OC |  |  | - |  | Absent |
| F/94 | ASM | 28 | 17 | - |  | Absent |
|  | OC |  |  | - |  | Absent |
| F/95 | ASM | 27 | 15 | - |  | Absent |
|  | OC |  |  | - |  | Absent |
| H/75** | ASM | 14 | 14 | - |  | / |
|  | OC | 0 |  | - |  | / |

* This cornea in the ASM group was used to image the macroscopic lesions after fluorescein staining.

** This pair of corneas was used for nerve mapping. Nerve detection was performed on the left eye directly after excision (PM = 9h30) and on the right eye after 14 days' organ culture storage and 14 days' rehabilitation in the Active Storage Machine.
